# A systematic review of prognostic factors at the end of life for people with a hematological malignancy

**DOI:** 10.1186/s12885-017-3207-7

**Published:** 2017-03-23

**Authors:** Elise Button, Raymond Javan Chan, Shirley Chambers, Jason Butler, Patsy Yates

**Affiliations:** 1 Royal Brisbane and Women’s Hospital, Bowen Bridge Road, Level 5, Block 34, Brisbane, QLD 4006 Australia; 2 Institute of Health and Biomedical Innovation, Queensland University of Technology, 60 Musk Avenue, Brisbane, QLD 4059 Australia; 3 Royal Brisbane and Women’s Hospital, Bowen Bridge Road, Level 5, Joyce Tweddell Building, Brisbane, QLD 4006 Australia; 4 Queensland University of Technology, Victoria Park Road, N Block, Brisbane, QLD 4059 Australia

**Keywords:** Hematological malignancies, Prognostic factors, End of life, Palliative care

## Abstract

**Background:**

Accurate prognosticating is needed when patients are nearing the end of life to ensure appropriate treatment decisions, and facilitate palliative care provision and transitioning to terminal care. People with a hematological malignancy characteristically experience a fluctuating illness trajectory leading to difficulties with prognosticating. The aim of this review was to identify current knowledge regarding ‘bedside’ prognostic factors in the final 3 months of life for people with a hematological malignancy associated with increased risk of mortality.

**Methods:**

A systematic review of the literature was performed across: PubMed; CINAHL; PsycINFO; and Cochrane with set inclusion criteria: 1) prognostic cohort studies; 2) published 2004–2014; 3) sample ≥ 18 years; 4) >50% sample had a hematological malignancy; 5) reported ‘bedside’ prognostic factors; 6) median survival of <3 months; and 7) English language. Quality appraisal was performed using the Quality In Prognostic Studies (QUIPS) tool. Results are reported in line with PRISMA guidelines.

**Results:**

The search returned 4860 studies of which 28 met inclusion criteria. Twenty-four studies were rated moderate quality, three were high quality and one study was deemed to be of low quality. Most studies were set in the ICU (*n* = 24/28) and were retrospective (*n* = 25/28). Forty ‘bedside’ prognostic factors were identified as associated with increased risk of mortality encompassing the following broad categories: 1) demographics; 2) physiological complications or conditions; 3) disease characteristics; 4) laboratory blood values; and 5) interventions.

**Conclusions:**

The literature on prognosticating in the final months of life was predominantly focused on people who had experienced acute physiological deterioration and were being treated aggressively in the in-patient setting. A significant gap in the literature exists for people who are treated less aggressively or are on a palliative trajectory. Findings did not report on, or confirm the significance of, many of the key prognostic factors associated with increased risk of mortality at the end of life in the solid tumour population, demonstrating key differences in the two populations.

**Trial registration:**

This systematic review was not registered.

**Electronic supplementary material:**

The online version of this article (doi:10.1186/s12885-017-3207-7) contains supplementary material, which is available to authorized users.

## Background

Hematological malignancies are a collection of heterogeneous neoplasms that are distinct from solid tumours largely by the presence of symptoms related to bone marrow failure or suppression [1]. These symptoms include bleeding, infection and anemia. The underlying disease and treatment of hematological malignancies can lead to episodes of acute deterioration that require intensive medical intervention [2]. Approximately 7% of people with a hematological malignancy admitted to hospital will become critically ill [3]. However, it is not uncommon for these patients to recover from close to death deterioration which can be successfully treated in many instances [4, 5]. People with a hematological malignancy characteristically experience a fluctuating illness trajectory [5] leading to difficulties with prognostication [6]. Often times, deterioration is unpredictable and rapid, and can lead to a swift change in goals of care from curative to palliative [7, 8].

Palliative care is a holistic approach that aims to improve quality of life for people with life-threatening illness [9]. Although traditionally viewed as terminal care, palliative care can be integrated at any time in an illness trajectory, alongside disease modifying and curative treatment [10]. In contrast, terminal care is provided as a patient nears the end of their life, however there is no agreed upon definition of the terminal phase in an illness [11]. It is agreed that the terminal phase is a time where goals of care should be focused on comfort, dignity and symptom management, rather than control of the cancer [11]. A growing body of literature has identified that significant challenges exist providing palliative care in the hematology setting [5]. Specifically, the unpredictable and fluctuating illness trajectory of people with a hematological malignancy and potential for rapid deterioration is reported to delay the identification and communication of transitions from a curative to a palliative focus of care [12]. These factors act as a barrier to timely palliative care integration in care and patients’ transition to the end of life [5]. Hematologists have reported difficulties in prognosticating as a hindrance to palliative care integration [6].

A prognosis is a prediction of possible future outcomes and is often focused on risk of mortality and time frames of survival [13, 14]. Predicting time frames of survival is difficult due to the complex nature of the human body and an increasing ability for medicine to alter the course of a disease [14]. This is particularly relevant in the hematology setting which has seen rapid developments in anti-cancer therapies (immunologic and targeted agents) in recent years [15]. Prognosticating near the end of life provides valuable information for people with a cancer diagnosis, their families and health care professionals [14, 16]. The question “how long do I have to live?” is one of the most important and daunting questions a person can ask their health care team [14]. Although palliative care is recommended to be integrated early and on a needs basis rather than based on prognostic information, accurate prognostic information allows health care professionals to know when to initiate open honest discussion with patients and their families about the end of life [14]. Ideally, conversations around potential deterioration and death should have already taken place earlier in the disease trajectory during times of clinical stability [17]. Honest discussions with patients who are nearing the end of life enable review of the goals of care, in line with the patient’s wishes, and clarification regarding the patient’s wishes for their health care management in the future, and at the end of life [18, 19]. Time for planning has been reported as an important issue for people living with advanced cancer [20]. Clinicians must be able to prognosticate accurately near the end of life in order to communicate effectively with patients and their family members, make informed decisions about treatment options, integrate palliative care when appropriate, and facilitate timing of referral to specialist palliative care services and planning for death [6, 14].

We conducted a systematic review of the literature to identify the prognostic factors in the final 3 months of life that are associated with increased risk of mortality for people with a hematological malignancy. The rationale for this review was to provide clinicians with an overview of current evidence regarding prognostic factors, to facilitate provision of palliative care as patients transition to the end of life. The review was also intended to identify gaps in the literature and guide future research.

## Methods

### Data sources

This review is reported in accordance with PRISMA guidelines on reporting reviews of the literature [21]. As per an a priori protocol, a systematic search of the literature was performed across the following electronic databases: a) PubMed; b) CINAHL c) PsycINFO; and d) Cochrane Central. WHO Clinical Trial Search Portal (www.who.int/trialsearch) was searched to identify relevant studies that are currently underway or have recently been completed and ProQuest Dissertation and Theses database was searched to identify grey literature. Additionally, reference lists of relevant papers were screened. Key words included: 1) hematological malignancy; 2) prognostic factors; and 3) end of life. Related synonyms and Medical Subject Headings (MESH) were utilised in the search. The protocol was not registered.

### Selection criteria

Decisions for inclusion of studies were made based on the selection criteria displayed in Table 1. Screening of search titles, abstracts and full texts was undertaken by a single person (EB). Challenging decisions regarding the inclusion of a paper were discussed with a second member of the research team (PY, RC or SC).

**Table 1:** Selection criteria

Inclusion criteria	Exclusion criteria
• English language only • Human research subjects with a hematological malignancy (>50% of study sample) • Adult patients (≥18 years of age) • Primary research • Peer reviewed articles • Studies from January 2004 to November 2014 • Prognostic studies (cohort or case-control) • Median survival of ≤ 3 months, (or >50% sample had died in ICU, or in hospital or within 3 months) • Reported ‘bedside prognostic factors’	• Editorials and letters • Discussions / expert opinion papers • Non - peer reviewed • Reports of clinical trials evaluating the effects of a specific treatment (chemotherapy, radiation therapy, etc.) • Systematic reviews of original research (predictive/prognostic cohort studies)

The search considered prognostic studies that examined predictive factors of mortality in people with a hematological malignancy in their final 3 months of life. Studies were included if the sample had a median survival of less than, or equal to, 3 months, or over 50% of the sample had died at 3 months. It is important to note that palliative care integration, review of goals of care and end-of-life care discussions and planning can occur at any time, not just in the final 3 months of life. During preliminary searching it was identified that studies regarding prognosticating at the end of life predominantly reported overall survival of no more than 3 months. This time period was chosen as it defined a homogeneous population and enhanced the ability to generalize results [11]. This time period has been used in other reviews reporting on prognosticating at the end of life [11, 22]. Additionally, this time period would be valuable in informing the transition to the end of life for people with a hematological malignancy. Studies in any health care setting or health care facility were included (i.e. at home in the community, palliative care unit, cancer care ward or intensive care unit). This wide inclusion criterion was utilised to provide a broad overview of the current scientific knowledge regarding prognosticating for people with a hematological malignancy at the end of life, and to highlight gaps in the literature. Research studies published within the last 10 years were included in the review due to the rapidly evolving nature of health care, specifically in the hematology setting. Studies were included if >50% of participants were people with hematological malignancies, or if the data reported were analysed independently in case-control studies comparing prognostic factors between people with solid tumours and hematological malignancies. All single cohort studies that met inclusion criteria (not comparison studies) included only participants with a hematological malignancy. Only studies reporting ‘bedside prognostic factors’ were included, meaning factors that are generally available and able to be assessed for in regular clinical practice by a range of clinicians, which include: performance and nutritional status; clinician estimates of survival; symptom burden; routine laboratory tests; quality of life; and socio-economic factors [11]. More complex prognostic factors that were excluded included cytogenetic tests and molecular markers as these require specialisation to interpret.

### Quality assessment

Quality appraisal was performed using the Quality In Prognostic Studies (QUIPS) tool as it supports a systematic appraisal of bias [23]. The QUIPS is an evidence-based, peer reviewed, practical system for assessing quality in prognostic studies by assessing bias in essential domains including: selection bias; confounding; outcome measurement; and prognostic factor measurement [23–25]. A formal quality appraisal tool with a scoring system was used to provide structure to the review however, the limitations of such a system are acknowledged [26]. Based on a recommendation from the Cochrane Collaboration, the main focus of quality assessment in this review was the individual features of the study rather than the quality appraisal score [26]. Hence, studies were included regardless of their quality appraisal score; limitations of the studies were also noted [26]. Each study was read in full and evaluated independently by a second member of the research team. Any divergent results were discussed between two members of the research team and a single rating was assigned.

### Analysis

A formal meta-analysis was not planned or attempted for this systematic review as the methods, populations, definitions or prognostic factors and results were too heterogeneous [27, 28]. The issue of integrating the results of prognostic studies is not unique to this review and has been found to be particularly problematic in prognostic studies due to inconsistent terminology, inadequate reporting and heterogeneity in study designs [29]. A narrative analysis was undertaken based on the statistical significance (*p* value) and Confidence Interval (CI) for each factor [11].

## Results

### Study selection

**Fig. 1:**
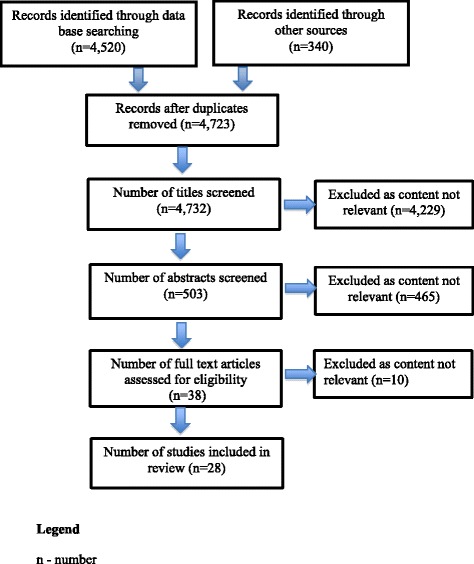
PRISMA Flow diagram for inclusion of studies

A search of the literature returned 4860 articles. After duplicates were removed, and titles and abstracts were screened against the set selection criteria, 37 studies were read in full, and 28 were found to be relevant and included in the review. See Fig. Fig. 1 for further details. No grey literature, or recently completed or ongoing studies, were identified.

### Study characteristics

Of the 28 studies included in the review, 24 investigated prognostic factors associated with increased risk of mortality in people admitted to the intensive care unit (ICU) [30–53]. One study explored prognostic factors in the context of intracranial hemorrhage in the hospital setting [54] and another included a sample of patients with invasive aspergillosis, also in hospital [55]. Two studies measured prognostic factors of palliative patients, one on referral to a specialist palliative care service (including in-patients and out-patients) [56], and the other on admission to a palliative care unit [57]. All but one of the included studies sampled participants from the in-patient setting, limiting the generalizability of findings to this population. Corbett et al. included a population in the out-patient and in-patient setting referred to a specialist palliative care service [56]. The studies were purely prognostic single cohort studies, with the exception of two. Hill et al. [49] and des Ordons et al. [44] employed prognostic case-control cohort designs and compared outcomes of people with a hematological malignancy admitted to the ICU against medical controls. The studies with a single cohort included people with a hematological malignancy or in comparison studies, only data on the group with a hematological malignancy were extracted and included in the results of this review. Including case-control studies in this manner did not impact the process of exploring prognostic factors and is not viewed as a limitation in regards to the aims of this review. Methodological features of the studies are presented in Table 2.

**Table 2:** Methodological features of studies

Author, Year, Country	Design	Sample characteristics	Hem malig	N	Analysis	Quality rating (QUIPS)
Rabe et al., (2004) Germany [41]	Retro	Admitted to ICU, with pulmonary infiltrates, requiring ventilation	AML	30	Uni -	Mod
Soubani et al., (2004) USA [30]	Retro	Admitted to ICU	HSCT	85	Uni Multi	Mod
BaHammam et al. (2005) Saudi Arabia [51]	Retro	Admitted to ICU	All	44	Uni -	Mod
Owczuk et al., (2005) Poland [37]	Retro	Admitted to ICU, requiring ventilation	HSCT	40	Uni Multi	Mod
Naeem et al., (2006) USA [34]	Retro	Admitted to ICU	Cord HSCT	44	Uni -	Mod
Ferra et al., (2006) Spain [46]	Retro	Admitted to ICU	All	100	Uni Multi	Mod
Lim et al., (2007) UK [33]	Retro	Admitted to ICU	All	55	Uni -	Mod
Yang et al., (2007) Taiwan [31]	Retro	Admitted to ICU, requiring ventilation	HSCT	41	Uni -	Mod
Nishida & Palalay, (2008) USA [36]	Retro	Admitted to ICU, requiring ventilation	All	37	Uni -	Mod
Park et al., (2008) Korea [38]	Retro	Admitted to ICU for septic shock	Acute leuk	50	Uni Multi	High
Chen et al., (2009) Taiwan [54]	Retro	Intracranial hemorrhage (in-patient acute setting)	AML	51	Uni Multi	High
Hampshire et al., (2009) UK [48]	Retro	Admitted to ICU (multi-centre)	All	7689	- Multi	Mod
des Ordons et al., (2010) USA [44]	Retro	Admitted to ICU	AML	45	Uni -	High
Burghi et al., (2011) France [53]	Retro	Admitted to ICU, invasive pulmonary aspergillosis, ventilated	All	67	Uni Multi	High
Depuydt et al., (2011) Belgium [43]	Pro	Admitted to ICU	Allo HSCT	44	Uni Multi	Mod
Geerse et al., (2011) Netherlands [47]	Retro	Admitted to ICU	All	86	Uni Multi	Mod
Park et al., (2011) Korea [39]	Retro	Admitted to ICU, with AKI, requiring RRT	All	94	Uni Multi	Mod
Ramos et al., (2011) USA [55]	Retro	Invasive aspergillosis (in-patient acute setting)	HSCT	449	Uni Multi	Mod
Agarwal et al. (2012) Australia [50]	Retro	Admitted to ICU	HSCT	146	Uni Multi	High
Ferra et al., (2012) Spain [45]	Retro	Admitted to ICU	Lymphoma	48	Uni Multi	Low
Hill et al., (2012) UK [49]	Retro	Admitted to ICU (multi-centre)	All	147	Uni Multi	Mod
Yeo et al., (2012) Korea [32]	Retro	Admitted to ICU	All	227	Uni Multi	Mod
Corbett et al., (2013) Australia [56]	Retro	Referred to specialist palliative care service (in-pt. & out-pt. setting)	All	276	- Multi	Mod
de Montmollin et al., (2013) France [42]	Retro	Admitted to ICU, with septic shock, pulmonary origin (multi-centre)	All	218	Uni Multi	Mod
Namendys-Silva et al., (2013) Mexico [35]	Pro	Admitted to ICU	All	102	Uni Multi	Mod
Price et al., (2013) USA [40]	Retro	Admitted to ICU, requiring ventilation	Acute leuk	167	Uni Multi	Mod
Kripp et al., (2014) Germany [57]	Pros	Admitted to palliative care unit	All	290	Uni Multi	Mod
Boyaci et al., (2014) Turkey [52]	Retro	Admitted to ICU	HSCT	48	Uni Multi	Mod

*AML* acute myeloid leukemia, *ICU* intensive care unit, *HSCT* hematopoietic stem cell transplant (allogeneic and autologous), *HM* hematological malignancy, *Cord HSCT* umbilical cord (source of stem cells) hematopoietic stem cell transplant, *alloHSCT* allogeneic hematopoietic stem cell transplant, *AKI* acute kidney injury, *RRT* renal replacement therapy, *HIV + ve* human immunodeficiency virus positive, *U* univariate statistical analysis / M - multi- multivariable statistical analysis

The majority of sample sizes were small, 21 of the 28 studies had a sample size of <150. Small sample size is a limitation of prognostic research [29] however, due to the specific nature of the cohort, it is acknowledged that a small sample may have been difficult to avoid. The majority of studies were retrospective (*n* = 25). Country of publication varied between the studies however they were primarily conducted in western developed countries that have similar health care systems. Most of the studies were conducted at a single centre, with the exception of three studies [42, 48, 49], which retrospectively reviewed data from multiple centres via electronic health databases. It is possible that results of single centre studies can be attributed only to the site where the research was conducted due to individual characteristics of that centre not present in other settings. Many studies had long periods of data collection; eleven studies collected data for 10 years or more [31, 37, 42, 45, 48, 50, 51, 53–55, 57]. Collecting data over long periods can lead to era bias, where changes in hospital practice and policy, or improved treatments and technologies can affect patient outcomes. Survival was measured via ICU mortality, hospital mortality, 30-day mortality and median survival time. Ideally, prognostic studies are best compared when there is similar reporting of survival outcomes [11].

### Quality of studies

Utilising the QUIPS tool, 24 studies were rated moderate quality, three were high quality and one study was deemed to be of low quality. All studies were included in the review regardless of their score and their limitations were noted. The quality appraisal score for each study is presented in Table 2.

### Patient characteristics

Collectively, the study samples had more male participants (61%) (range 33% [30] to 78% [50]). Participants’ age ranged from a mean of 32.7 years (SD 8.8) [31] to a median of 70.5 years (range 15–98) [57]. The latter of the ranges describes a sample from a palliative care unit which may explain the older population. Only one study reported the race of the sample, which was: 82% white; 11.7% African American; and 5% other [30]. No study discussed patients’ socio-economic status or education level. Fourteen studies included all types of hematological malignancies [32, 33, 35, 36, 39, 42, 46–49, 51, 53, 56, 57]; five sampled patients with acute leukemia [38, 40, 41, 44, 54]; eight looked at people who had undergone hematopoietic stem cell transplantation [30, 31, 34, 37, 43, 50, 52, 55]; and one was specifically focused on people with human immunodeficiency virus associated lymphoma [45]. The patient population was predominantly treated with aggressive curative or life-prolonging intent, as most studies (*n* = 24) had admission to the ICU as a criterion for inclusion. [58].

### Analysis in studies

Seven of the 28 studies did not conduct multivariable analysis [31, 33, 34, 36, 41, 44, 51], and therefore did not account for the effect of multiple explanatory variables on the variable of interest, limiting the results [59, 60]. This is likely due to the fact that many study samples were small and had a limited number of events, i.e., deaths in the context of this review. Due to limitations associated with small sample sizes and low numbers of events, prognostic factors identified in univariate and multivariable analysis were included in the results of this review. An extensive range of prognostic factors were measured in the 28 studies. Many of the studies tested more factors than merited by their small sample size. As a general rule, at least 10 events are needed for each prognostic factor to allow for multivariable regression analysis [61, 62]. In addition, of the 21 studies that used multivariable analyses, goodness of fit testing was reported in only seven studies. Williams et al. [63] report that ‘over fitted’ models with no description of model validation are a key weakness in prognostic studies.

### Prognostic factors identified

A wide range of variables were tested as potential prognostic factors in the studies. Many of these factors were described inadequately or measured via different methods. Collectively, 40 bedside prognostic factors were reported to be significantly associated with increased risk of mortality in people with a hematological malignancy. These prognostic factors were conceptualized into the following categories: demographic; interventions; physiological complications or conditions; disease characteristics; and laboratory blood values. Between the studies, certain variables measured similar concepts, and were therefore aggregated under a single prognostic factor for ease of analysis. This occurred for sepsis/infection, coagulopathy, liver dysfunction, elevated liver enzymes, elevated creatinine or urea, respiratory distress/failure, decreased level of consciousness (LOC), cardiovascular function and hemodynamic instability. Prognostic factors identified as associated with increased risk of mortality in univariate and/or multivariable analyses are presented in Additional file 1. Attributes of the prognostic factors identified as significantly associated with increased risk of mortality are presented in Table 3.

**Table 3:** Attributes of commonly reported prognostic factors

Prognostic factor	Number of studies	Total # of pts., N	Univariate analysis Significant/ tested/% significant	Multivariable analysis Significant/ tested/% significant	Hematological malignancies tested
Older age	22	10,027	6/20 (30%)	5/16 (31%)	• Acute leukemia • All hematological malignancies • AML • Cord HSCT • HSCT
Mechanical ventilation	18	9702	13/17 (76%)	12/14 (86%)	• Acute leukemia • All hematological malignancies Cord HSCT • HSCT • Lymphoma
Vasopressors use	15	1300	12/15 (80%)	8/9 (89%)	• Acute leukemia • All hematological malignancies • AML • Cord HSCT • HSCT
Renal replacement therapy	7	633	3/7 (43%)	3/4 (75%)	• All hematological malignancies • AML • HSCT
Transfusions	4	490	2/4 (50%)	2/4 (50%)	• Acute leukemia • All hematological malignancies • HSCT
Admission to intensive care unit	1	85	1/1 (100%)	1/1 (100%)	• HSCT
Artificial feeding	1	290	1/1 (100%)	1/1 (100%)	• All hematological malignancies
Opiate analgesia	1	290	1/1 (100%)	1/1 (100%)	• All hematological malignancies
Sepsis/infection	17	9170	9/16 (56%)	8/14 (57%)	• Acute leukemia • All hematological malignancies • AML • HSCT • Lymphoma
Hemodynamic instability	9	8095	6/8 (75%)	5/8 (62%)	• All hematological malignancies • AML • Cord HSCT • HSCT • Lymphoma
Multi organ failure	9	657	6/8 (75%)	5/6 (83%)	• All hematological malignancies • HSCT
Respiratory distress/failure	13	8715	5/12 (42%)	5/10 (50%)	• Acute leukemia • All hematological malignancies AML • Cord HSCT • HSCT • Lymphoma
Cardiovascular function	5	711	3/5 (60%)	3/5 (60%)	• All hematological malignancies • HSCT • Acute leukemia
Decreased level of consciousness	10	1169	3/9 (33%)	3/8 (37%)	• Acute leukemia • All hematological malignancies • HSCT • Lymphoma
Renal dysfunction	8	8205	2/6 (33%)	2/3 (66%)	• Acute leukemia • All hematological malignancies • AML • Cord HSCT • HSCT • Lymphoma
Fungal infection	4	382	2/4 (50%)	2/4 (50%)	• Acute leukemia • All hematological malignancies • HSCT
Liver dysfunction	7	478	3/7 (43%)	3/4 (75%)	• Acute leukemia • AML • Cord HSCT • All hematological malignancies • HSCT
Pneumonia	4	173	2/4 (50%)	0/1 (0%)	• All hematological malignancies • Cord HSCT • HSCT
CMV reactivation	1	86	1/1 100%	0/1 0%	• All hematological malignancies
Performance status	4	775	1/4 (25%)	1/4 (25%)	• Acute leukemia • All hematological malignancies
Disease characteristics					
Acute leukemia	11	9352	2/10 (20%)	4/10 (40%)	• All hematological malignancies • Cord HSCT • HSCT
Relapse or advanced disease	10	862	4/10 (40%)	4/8 (50%)	• Acute leukemia • All hematological malignancies • AML • Cord HSCT • HSCT
HSCT	9	8617	1/8 (12%)	1/7 (14%)	• All hematological malignancies • HSCT
Laboratory values					
Liver enzymes	11	823	7/11 (64%)	5/6 (83%)	• Acute leukemia • All hematological malignancies • AML • HSCT
Urea or creatinine	11	8294	5/10 (50%)	3/7 (43%)	• Acute leukemia • All hematological malignancies • AML • Cord HSCT • HSCT
Neutropenia or leukopenia	15	9244	5/14 (36%)	5/12 (42%)	• All hematological malignancies • AML • HSCT
Thrombocytopenia	10	8614	5/9 (55%)	4/6 (67%)	• All hematological malignancies • AML • Cord HSCT • HSCT
Coagulopathy	8	512	4/7 (57%)	3/4 (75%)	• Acute leukemia • All hematological malignancies • AML • HSCT • Lymphoma
Anemia	4	477	4/4 (100%)	4/4 (100%)	• All hematological malignancies • HSCT
Blood pH	3	7960	2/3 (66%)	2/2 (100%)	• All hematological malignancies
Calcium	2	338	2/2 (100%)	2/2 (100%)	• All hematological malignancies • HSCT
CRP	3	368	2/3 (66%)	2/2 (100%)	• All hematological malignancies • AML • HSCT
Hematocrit	4	7859	1/3 (33%)	2/4 (50%)	• All hematological malignancies
Hypoalbuminemia	5	570	2/5 (40%)	2/4 (50%)	• All hematological malignancies • AML • HSCT
Lactate	2	133	2/2 (100%)	2/2 (100%)	• HSCT
LDH	2	7737	2/2 (100%)	2/2 (100%)	• All hematological malignancies • HSCT
Sodium	2	338	½ (50%)	2/2 (100%)	• All hematological malignancies • HSCT
Bicarbonate	1	48	1/1 (100%)	1/1 (100%)	• HSCT
Pro-calcitonin	1	48	1/1 (100%)	1/1 (100%)	• HSCT
Uric acid	1	48	1/1 (100%)	1/1 (100%)	• HSCT

N.B. Only studies which reported on the prognostic indicator are included in the univariate and multivariable analysis reporting

N.B Prognostic factors that had a large overall sample were predominantly made up of the numbers from Hampshire et al. [48] (*n* = 7689)

*HSCT* Hematopoietic stem cell transplant/*AML* acute myeloid leukemia

### Demographic

Age was the most commonly studied variable however, it did not appear to be of prognostic importance. In the 22 studies that investigated this factor, only 30% found increasing age was significantly associated with increased risk of mortality in both univariate and multivariable analyses, in a combined sample of 10,027 people.

### Interventions

In terms of interventions, aggressive therapies associated with critical illness were the most consistent prognostic factors of increased risk of mortality. Receiving vasopressor support was predictive of, or associated with, increased risk of mortality in 89% of multivariable analyses models and 80% of univariate testing in the 15 studies that measured this variable (total sample *n* = 1300). Similarly, receiving mechanical ventilation was predictive of mortality in 86% of multivariable analyses modelling and 76% of univariate testing in 18 studies (total sample *n* = 9702). Receiving renal replacement therapy in the ICU was also identified as a significant prognostic factor in 43% of univariate results (*n* = 3/7) and in 75% of multivariable results (*n* = 3/4) in a total sample of 633 participants.

Ramos and colleagues [55] identified that admission to an ICU was significantly associated with increased risk of mortality. This was the only study to identify this prognostic factor however this may be because this study was one of few studies that did not examine prognostic factors of people already admitted to the ICU. Only one study investigated receiving opiate analgesia and artificial feeding as prognostic factors, and this study was one of the two studies conducted in a palliative setting [57].

### Physiological complications or condition

Under the category of physiological complications or conditions, prognostic factors related to serious illness and bone marrow failure or suppression was consistently associated with worse survival or was predictive or mortality. Multi-organ failure was associated with increased risk of mortality in 75% of univariate analyses and 83% of multivariable analyses in the 9 studies that investigated this factor (total sample *n* = 657). Hemodynamic instability was tested in 9 studies, total sample of 8095, and was significant in 75% of univariate results (*n* = 6/8) and 62% of multivariable results (*n* = 5/8). Liver failure, renal failure and cardiac failure were significantly correlated with decreased survival. Symptoms related to bone marrow failure or suppression, including anemia and coagulopathy, were also predictive of mortality. Sepsis or the presence of infection appeared to be predictive of mortality but the magnitude of this association was moderate: 56% in univariate testing and 57% in multivariable modelling. The findings of this review suggest limited value in performance status as a prognostic factor, with only one of the four studies which tested this variable finding it to be statistically significant. No study included in this review reported on clinician judgement, quality of life, comorbidities and symptom burden.

### Disease characteristics

Disease stage (relapsed/advanced disease) did not appear to be associated with survival and was significant in 40% of studies that tested this factor (*n* = 4/10). A diagnosis of leukemia was investigated as a prognostic factor in 11 studies (total sample *n* = 9352) and was significant in univariate results in 20% of studies (*n* = 2/10) and in multivariable results in 40% of studies (*n* = 4/10). A history of hematopoietic stem cell transplant also did not appear to be associated with increased risk of mortality and was significant in only 12% of univariate results (*n* = 1/8) and 14% of multivariable results (*n* = 1/7) in a total sample of 8617 people.

### Laboratory blood values

Laboratory blood values related to bone marrow suppression or failure and active cancer such as neutropenia, thrombocytopenia, anemia and coagulopathy were moderately associated with increased risk of mortality. Abnormal blood PH, elevated calcium, elevated liver enzymes and elevated urea and creatinine were also predictive of mortality. Hypoalbuminemia did not appear to be associated with increased risk of mortality in the 5 studies which investigated this prognostic factor [30, 35, 44, 52, 57], and was significant in 40–50% of results (*n* = 2/5 univariate and *n* = 2/4multivariable) with a total sample of 570 participants. A range of other factors was tested with varying results (see Table 3 and Additional file 1 for further details of prognostic factors).

## Discussion

### Prognostic factors

The aim of this review was to identify the current evidence regarding prognosticating in the final 3 months of life for people with a hematological malignancy, for the purpose of informing provision of palliative care as patients transition to the end of life. This review also aimed to identify gaps in the literature and guide future research. Not surprisingly, our findings substantiate previous research that demonstrates critical illness and the need for aggressive life supporting measures are associated with increased risk of mortality for patients with a cancer diagnosis [64, 65]. Receiving mechanical ventilation and vasopressor support were among the strongest prognostic factors for people with a hematological malignancy in the ICU. As is found in the solid tumor population, multi-organ failure, organ failure (liver, renal or cardiac) and hemodynamic instability were associated with worse survival for people with a hematological malignancy.

The studies included in our review did not report on, or confirm the significance of, many of the key prognostic factors associated with increased risk of mortality at the end of life in the solid tumour population. This includes prognostic factors such as performance status, presence of co-morbidities, symptom burden, quality of life, disease stage or symptoms of anorexia-cachexia syndrome [11, 66]. This is likely to be partially due to the fact that these factors were not adequately tested in the studies in this review, which were largely focused on prognostic factors routinely measured in the ICU setting in the context of acute deterioration. This could also be partly due to the fact that the patient cohort in many of the studies included in this review were dying from acute critical illness in contrast to slower, more predictable disease progression as commonly seen in the solid tumour population [67]. Sudden deterioration to a terminal event is reported to be a common occurrence in the hematology setting leading to issues with prognosticating and the provision of palliative care [6]. Prognostic factors that are only present in the context of acute deterioration and critical illness (i.e. mechanical ventilation and vasopressor support) are not likely to be useful in facilitating effective and timely integration of palliative care, as they are present too late in the illness trajectory. However, they are useful for identifying the appropriate time to transition the care of a patient with a hematological malignancy to align with a palliative approach as they enter the terminal phase.

Several palliative prognostic tools exist, predicting time frames of survival and risk of death for people with chronic illness and solid tumors as they near the end of life. However, it is unclear if these tools are applicable to the specific illness trajectory experienced by people with a hematological malignancy [68]. A number of studies exist highlighting key prognostic factors in the final months of life for people with a solid tumor diagnosis. It is also unclear if these factors can inform palliative care provision and transitioning of patients to the end of life in the hematology setting, due to the significant gap in the literature highlighted in this review [11]. Disease progression for certain hematological malignancies may be more closely aligned to trajectories of people with organ failure and advanced non-oncological diseases such as respiratory and heart failure or human immunodeficiency virus and acquired immune deficiency syndrome [69]. For these patients, the illness trajectory is gradual and marked with episodes of acute deterioration which may lead to death, or be followed by some recovery. In this trajectory, death is sudden and seemingly unexpected [70]. In this clinical scenario it is important to integrate palliative care and plan for potential palliative needs early for at-risk patients, prior to acute and rapid deterioration. It is acknowledged the clinical course of people with hematological malignancies varies significantly between disease types and within an individual disease depending on a range of factors [4]. It is possible that some people with a hematological malignancy, particularly those with less aggressive disease, will share a similar trajectory to people with solid tumours, while others will not. The differences in illness trajectories should impact on the timing and manner in which palliative care is integrated and provided [70]. It is likely that all people with a hematological malignancy have been treated in the same manner and models of care and in which their solid tumour counterparts are treated, negatively impacting on palliative care integration and provision [71]. Further research is needed to address these unanswered questions and inform clinical practice.

Quality of life and symptom burden was not assessed in the included studies, despite these factors being associated with increased risk of mortality at other times in the illness trajectory for people with a hematological malignancy, and in people with other types of malignancies [11, 66, 72, 73]. It is acknowledged that these factors are difficult to assess retrospectively, as was the methodology of most studies. The majority of the studies in this review did not assess the effect of performance status and number of co-morbidities despite these factors also being reported as predictive of survival for people with a hematological malignancy at other stages in the illness trajectory; for example prior to stem cell transplantation [2, 74]. Surprisingly, none of the studies investigated clinician judgement (the clinicians’ prediction of survival) which, despite having limitations, is still considered to be useful in clinical practice, especially when used in combination with known prognostic factors and/or tools [22, 75]. Furthermore, the widely used surprise question “would you be surprised if this patient died in the next 12 months” [16] has not been tested in the hematology setting to the best of our knowledge. The body of literature identified in this review, did not adequately test all potentially relevant prognostic factors for people with a hematological malignancy in the final 3 months of life, leading to significant gaps in knowledge.

### Unique population of ‘terminal ill’ patients

While there are no standard criteria for defining the ‘end of life’ or ‘terminal phase’, a median survival of 3 months or less is often used to identify a terminal population [11]. Despite being close to death, most of the study populations did not appear to meet many of the criteria often used to described people in the terminal phase, such as: being recognised as approaching death; having ‘exhausted’ all curative treatment options; evidence of progressive malignant disease; or having goals redefined and focused towards symptom management [11]. People with a hematological malignancy often do not present as ‘terminally ill’ patients in their final months of life; they appear to have a shorter terminal phase compared to people with a solid tumor diagnosis. Therefore, current definitions and notions of the ‘terminal phase’ may not be suited to people with a hematological malignancy in regards to the provision of palliative care and end-of-life care.

The body of literature found in this review supports the notion that people with a hematological malignancy are reported to have a fluctuating illness trajectory, deteriorate rapidly to a terminal event and receive intense medical interventions often close to death [5, 76, 77]. It is difficult to establish the futility of aggressive interventions for people with a hematological malignancy as the absolute absence of benefit is often hard to identify and may be dependent on the particular person and their family [58]. However, the utility verse harm associated with such interventions should be determined. A person’s quality of life between episodes of acute illness must also be taken into consideration when considering palliative care integration and provision. The literature reflects that people with a hematological malignancy experience significant symptom burden near the end of life [78]. Due to this cohort’s fluctuating and unpredictable illness trajectory, identification of risk of deteriorating and dying may be more useful than predicting time frames for survival. The best way to provide people with a hematological malignancy with effective palliative and end-of-life care may be to: a) identify those who are at risk of deteriorating and dying [16]; and b) integrate palliative care alongside curative or life prolonging care, which is recommended in the hematology setting [10, 16].

Identifying people with progressive chronic illness who are at risk of dying, as a means of highlighting potential palliative needs, is a concept that is gaining momentum, and has become key a strategic policy in many health care services [79, 80]. This concept is largely unexplored in the hematology setting. Research is needed to explore this concept and the clinical indicators that are associated with risk of deteriorating and dying for people with a hematological malignancy, prior to acute deterioration. It is argued that too much emphasis is placed on predicting mortality and time frames of survival and too little is placed on planning for potential palliative care needs [81]. Future research needs to study patients with a hematological malignancy in all health care settings (in-patient and out-patient, acute and non-acute) prior to acute physiological deterioration. Additionally, studies are needed to investigate the impact of identifying at-risk patients and the notion of integrating and providing palliation along-side curative or life-prolonging care [5].

The literature reports there are challenges associated with integrating, and the provision of, palliative care in the hematology setting [5]. This includes, but is not limited to, the fluctuating and unpredictable illness trajectory experienced by many people, unclear goals of care in the context of new therapies and clinical trials, and a lack of knowledge of palliative care amongst, patients, families and health care professionals [5]. Additionally, the explosion of new therapies (immunologic and targeted agents) in the hematology setting is altering the disease course of many malignancies leading to further difficulties in prognosticating [15]. Previous research has demonstrated benefits associated with early integration of palliative care services for people with life-threatening illness (i.e. referral to specialist palliative care service on diagnosis of malignancy) [82, 83]. To the best of our knowledge, the effects of early palliative care integration have not yet been studied rigorously in the hematology setting in interventional studies. Studies exploring Advance Care Planning prior to stem cell transplantation reported it was associated with benefits for the patient and their carer, and was not associated with reduced survival [84–86]. These studies were observational in nature therefore results must be viewed with caution. However, the findings demonstrate that discussions and planning for the end of life can be successfully provided alongside aggressive treatment of life-prolonging or curative intent in the hematology setting.

### Lack of focus on palliative care

Studies with a palliative care focus, to inform this review, were scant within the literature. None of the included studies set in the ICU or acute care setting discussed identifying palliative care needs or palliative care integration or provision, despite their sample having a high mortality rate. It is acknowledged that this was not the aim of the included studies.

Of interest, during preliminary searching a large body of literature was found regarding prognosticating in the hematology setting that is focused on the earlier stages in the disease trajectory, such as upon diagnosis or pre-stem cell transplantation. Pre-stem cell transplantation, the presence and number of co-morbidities appears to be predictive of mortality and the Hematopoietic Stem Cell Specific Comorbidity Index has been developed for use [74]. Prognosticating for patients with a newly diagnosed hematological malignancy focuses on prognostic factors such as age, stage of disease, histology, and receptor status [2]. A number of prognostic tools exist for certain hematological malignancies upon diagnosis, including but not limited to, the International Prognostic Scoring System and the International Prognostic Indicator and related versions [2]. However, as the disease advances and the person’s physical condition deteriorates, issues such as malnutrition, poor performance, bleeding and infection are reported to be associated with the end of life for people with a hematological malignancy [57]. These factors have not been adequately explored in the literature. The study by Kripp and colleagues’ investigating prognostic factors of people admitted to a palliative care unit was the only study to test a range of prognostic factors traditionally associated with advanced illness, such as opiate use, requiring artificial feeding, poor performance and hypoalbuminemia [57]. Additionally, this study investigated prognostic factors associated with signs of end-stage hematological malignancies including anemia, thrombocytopenia and requiring transfusion support [76, 87, 88]. It is difficult to establish the significance of prognostic factors that were tested in few studies. A significant gap in the literature exists regarding prognostic factors that are relevant outside the ICU, prior to acute physiological deterioration.

### Limitations

A major limitation of this review was a lack of variation in study settings and designs. The studies identified in this review were predominantly focused on people being treated aggressively in the in-patient setting, particularly in the ICU. It is not clear if any of the prognostic factors identified in these studies are transferable to the broader hematology population. Most studies (*n* = 25) employed a retrospective design and were therefore only able to assess prognostic factors that were routinely collected in clinical practice for association with increased risk of mortality. The lack of variation in study settings and samples in this body of literature significantly limits the generalisability of findings. However, the aim of the review was to identify the current knowledge base regarding prognosticating in the final 3 months of life, identify gaps in knowledge and guide future research. As the majority of studies were set in the ICU (*n* = 24) only prognostic factors that are routinely measured on admission to, or in this setting, could be assessed. This led to significant bias of the potential prognostic factors studied. Clinical practice and the type of data collected in the ICU are often very different to that of a ward, out-patient department, palliative care unit, or in the community. Only two studies investigated prognosticating for people treated with palliative intent [56, 57]. Another limitation of the review was the heterogeneity of the prognostic factors tested and the measurements utilised in studies which made the collation and comparisons across study findings difficult. In addition, differences in statistical analyses and varying end-points for survival have complicated the clinical applicability of findings [11].

Our review had some methodological limitations. It is acknowledged that the inclusion criterion for survival times excluded more recent studies set in the ICU, in which survival rates for people with a hematological malignancy have improved in recent years. However, the focus of this review was to identify prognostic factors for people who were nearing the end of their life therefore this inclusion criterion was considered practical and appropriate. Similar criteria has been previously used in similar reviews exploring survival prediction at the end of life [11, 22]. Additionally, non-English language studies were excluded. It is possible that some potentially relevant studies were missed however it is unlikely that papers presenting the best available evidence were overlooked as the prominent international hematology journals are published in English. Also, a small selection of key databases was searched for this review. It is unlikely that any noteworthy studies were missed in database searching, as this was supplemented by Google Scholar searching and hand searching via screening reference lists of relevant papers.

Overall, the quality of the articles was of a moderate to high standard, with only one study graded as low evidence as per the QUIPS tool. The clinical significance of many of the prognostic factors identified may be hindered by methodological flaws in the studies including: a) small sample size; b) single centre; c) measurement of prognostic factors varied between studies; d) lack of adjustment for important prognostic factors such as performances status; e) different reporting of survival end-points; and f) the retrospective nature of most studies [11]. Certain prognostic factors identified as significant in univariate analysis, were not identified as independently associated with increased risk of mortality in multivariable regression modelling, thus limiting the results of such findings. Results must also be viewed with caution as many studies tested more potential prognostic factors than warranted by their small sample size. Additionally, none of the studies described the admission policy to the ICU or palliative care unit the study was set in, or referral policy to the specialist palliative care unit. Therefore it was difficult to establish if study samples were representative of a larger population and if results are generalizable.

### Unanswered questions

Our objective was to identify the key prognostic factors associated with increased risk of mortality in the final 3 months of life for people with a hematological malignancy to inform palliative care provision as patients transition to the end of life. Gaps in the body of knowledge have also been identified to guide future research. Due to significant limitations in the findings, the results are not likely to be useful in clinical decision-making; rather, the findings highlight many unanswered questions and serve to inform future research. The lack of focus on prognosticating near the end of life, identifying risk of deteriorating and dying, provision of palliative care, and transitioning to the end of life for people with a hematological malignancy, supports the need for more research in this area [22]. The best way forward may be to investigate clinical indicators which identify risk of deteriorating and dying, rather than prognostic factors that predict time frames of survival due to the unpredictable nature of hematological malignancies. Study samples must include people prior to acute deterioration and in a range of settings. Studies are needed that are prospective, multi-centred, have large sample sizes and are methodologically rigorous in multivariable testing. Further research is needed investigating the importance of clinical indicators such as performance status, symptoms, clinician prediction (specifically the surprise question) and potentially quality of life.

## Conclusion

The body of literature reporting prognostic factors at the end of life for people with a hematological malignancy, is predominantly focused on people who have already deteriorating and are receiving aggressive treatment. Due to the unpredictable and fluctuating illness trajectory experience in this population, research is needed to identify risk of dying, prior to acute physiological deterioration, to improve palliative and end-of-life care delivery. Exact survival times cannot be predicted with certainty, particularly in this population. However, identifying people who have limited time remaining or are at risk of dying can enable clinicians to provide quality care, and allow patients and families the opportunity to plan for the future and have their wishes known.

## Additional file

Additional file 1: Prognostic factors identified as associated with mortality. In Additional file 1, the findings for each prognostic factor are displayed for each study. If a prognostic factor was tested in a study, the results are reported for that factor (i.e. if the factor was significant and the direction of association). It is also recorded if results were not reported. (DOCX 29 kb)

## Abbreviations

AKI: Acute kidney injury
alloHSCT: Allogeneic hematopoietic stem cell transplant
AML: Acute myeloid leukemia
Cord HSCT: Umbilical cord (source of stem cells) hematopoietic stem cell transplant
Fail: Failure
HIV + ve: Human immunodeficiency virus positive
HM: Hematological malignancy
HSCT: Hematopoietic stem cell transplant (allogeneic and autologous)
ICU: Intensive care unit
Leuk: Leukemia
LOC: Level of consciousness
M: Multi- multivariable statistical analysis
MESH: Medical Subject Headings
nr: Level of significance not reported
ns: Not significant
QUIPS: Quality In Prognostic Studies tool
Resp: Respiratory
RRT: Renal replacement therapy
U: Univariate statistical analysis
