# Supplementary material for: A systematic review of prognostic factors at the end of life for people with a hematological malignancy
Source: BMC Cancer. 2017 Mar 23;17:213. doi: 10.1186/s12885-017-3207-7 (PMC5364562; doi:10.1186/s12885-017-3207-7)
Supplement: Additional file 1: — Prognostic factors identified as associated with mortality. In Additional file 1, the findings for each prognostic factor are displayed for each study. If a prognostic factor was tested in a study, the results are reported for that factor (i.e. if the factor was significant and the direction of association). It is also recorded if results were not reported. (DOCX 29 kb) [file 12885_2017_3207_MOESM1_ESM.docx]

**ADDITIONAL FILES**

**Additional file 1. Prognostic factors identified as associated with mortality**

| Prognostic factors | Rabe, et al. (2004). | Soubani, et al. (2004). | BaHammam, et al. (2004). | Owczuk, et al. (2004). | Naeem, et al. (2006). | Ferra, et al. (2007). | Lim, et al. (2006). | Yang, et al. (2006). | Nishida, et al. (2008). | Park, et al. (2008). | Chen, et al. (2009). | Hampshire, et al. (2009). | des Ordons, et al. (2010). | Burghi, et al. (2011). | Depuydt, et al. (2011). | Geerse, et al. (2011). | Park, et al. (2011). | Ramos, et al. (2011). | Agarwal, et al. (2012). | Ferra, et al. (2012). | Hill, et al. (2012). | Yeo, et al. (2012). | Corbett, et al. (2013). | de Montmollin, et al. (2013). | Namendys-Silva, et al. (2013). | Price, et al. (2013). | Kripp, et al. (2014). | Boyaci, et al. (2014). | **Total studies significant** |
| --- | --- | --- | --- | --- | --- | --- | --- | --- | --- | --- | --- | --- | --- | --- | --- | --- | --- | --- | --- | --- | --- | --- | --- | --- | --- | --- | --- | --- | --- |
| **Demographic** | | | | | | | | | | | | | | | | | | | | | | | | | | | | |  |
| Older age | **+** | **ns** |  | **ns** | **ns** |  | **ns** | **+** | **ns** | **ns** | **-** | **+** | **+** | **ns** |  | **ns** | **ns** | **ns** | **ns** |  | **+** | **ns** | **+** | **+** | **ns** |  |  | **ns** | **8** |
| **Interventions** |  |  |  |  |  |  |  |  |  |  |  |  |  |  |  |  |  |  |  |  |  |  |  |  |  |  |  |  |  |
| MV |  | **+** | **ns** |  | **ns** | **+** | **ns** |  |  | **+** |  | **ns** | **+** |  |  | **+** |  | **+** | **ns** | **+** | **+** | **+** |  | **+** | **+** | **+** |  | **+** | **13** |
| Vasopressors use | **ns** |  | **ns** | **+** | **+** |  | **+** |  | **+** | **+** |  |  | **+** |  |  | **+** |  |  |  |  | **+** | **+** |  | **+** | **+** | **ns** |  | **+** | **12** |
| RRT |  |  | **ns** |  |  |  |  |  | **ns** |  |  |  | **ns** |  |  |  | **+** |  |  |  | **ns** |  |  | **+** |  |  |  | **+** | **2** |
| Transfusions |  |  |  |  |  |  |  |  |  | **ns** |  |  |  |  |  |  |  |  |  |  |  |  |  |  | **ns** |  | **+** | **+** | **2** |
| Admission to ICU |  | **+** |  |  |  |  |  |  |  |  |  |  |  |  |  |  |  |  |  |  |  |  |  |  |  |  |  |  | **1** |
| Artificial feeding |  |  |  |  |  |  |  |  |  |  |  |  |  |  |  |  |  |  |  |  |  |  |  |  |  |  | **+** |  | **1** |
| Opiate analgesia |  |  |  |  |  |  |  |  |  |  |  |  |  |  |  |  |  |  |  |  |  |  |  |  |  |  | **+** |  | **1** |
| **Physiological complications or conditions** | | | | | | | | | | | | | | | | | | | | | | | | | | | | |  |
| Sepsis/infection |  | **+** |  | **ns** |  | **+** |  | **+** | **ns** |  | **ns** | **+** | **+** | **+** | **-** |  |  |  | **ns** | **nr** | **+** | **ns** |  |  | **ns** | **ns** |  | **+** | **9** |
| Hemodynamic instability |  |  | **ns** | **+** | **ns** | **+** |  |  | **+** |  |  | **+** | **+** |  |  |  |  |  |  | **+** |  |  |  |  |  |  |  | **+** | **7** |
| Multi organ failure |  | **+** | **ns** | **ns** |  | **+** | **nr** |  | **+** |  |  |  |  |  |  |  |  |  | **+** |  |  |  |  |  | **+** |  |  | **+** | **6** |
| Resp distress/fail | **ns** | **ns** |  |  | **ns** |  |  | **+** | **ns** |  |  | **+** |  | **ns** |  | **ns** |  |  | **+** | **+** |  | **ns** |  |  |  | **+** |  | **+** | **6** |
| Cardiovascular function |  | **ns** |  |  |  |  |  |  |  |  |  |  |  |  |  | **ns** |  |  | **+** |  |  | **-** |  |  |  | **+** |  |  | **3** |
| Decreased LOC |  | **ns** | **ns** |  |  | **+** |  |  |  |  |  |  |  |  |  | **ns** |  |  | **+** | **nr** |  | **ns** |  | **+** |  | **ns** |  | **ns** | **3** |
| Renal dysfunction | **ns** |  | **ns** |  | **ns** |  |  |  | **+** |  |  | **+** |  |  |  |  |  |  | **+** | **nr** |  |  |  |  |  | **ns** |  |  | **3** |
| Fungal infection |  |  |  |  |  | **+** |  |  |  | **ns** |  |  |  |  |  | **ns** |  |  | **+** |  |  |  |  |  |  |  |  |  | **2** |
| Liver dysfunction | **ns** |  |  |  | **ns** | **+** |  | **ns** |  |  |  |  |  |  |  |  |  |  |  | **+** |  |  |  |  |  | **ns** |  | **+** | **2** |
| Pneumonia |  |  | **ns** |  | **+** |  |  |  | **+** |  |  |  |  |  |  |  |  |  |  |  |  |  |  |  |  |  |  | **ns** | **2** |
| CMV reactivation |  |  |  |  |  |  |  |  |  |  |  |  |  |  |  | **+** |  |  |  |  |  |  |  |  |  |  |  |  | **1** |
| Performance status |  |  |  |  |  | **ns** |  |  |  |  |  |  |  |  |  |  |  |  |  |  |  |  |  | **ns** |  | **ns** | **+** |  | **1** |
| **Disease characteristics** | | | | | | | | | | | | | | | | | | | | | | | | | | | | |  |
| Acute leukemia |  |  |  |  | **ns** |  |  |  |  |  |  | **+** |  | **ns** |  | **ns** |  | **+** | **ns** |  |  | **+** | **+** | **ns** | **ns** |  |  | **ns** | **4** |
| Relapse/advanced |  |  | **+** |  | **ns** | **ns** |  |  |  | **+** | **ns** |  | **ns** |  |  | **ns** |  |  |  |  |  | **ns** |  |  |  | **+** |  | **+** | **4** |
| HSCT |  |  |  | **ns** |  | **ns** | **ns** |  | **ns** |  |  | **+** |  | **ns** |  | **ns** | **+** | **ns** |  |  |  |  |  |  |  |  |  |  | **2** |
| **Laboratory blood values** | | | | | | | | | | | | | | | | | | | | | | | | | | | | |  |
| Liver enzymes | **ns** | **ns** | **+** |  |  | **+** | **+** |  | **ns** | **+** |  |  | **ns** |  |  |  |  |  |  |  |  | **+** |  |  | **+** |  |  | **+** | **7** |
| Urea or creatinine |  | **ns** | **ns** |  | **+** | **+** |  |  | **ns** | **+** |  | **+** | **ns** |  |  |  |  |  |  |  |  | **ns** |  |  | **+** |  |  | **+** | **6** |
| Neutro/leukopenia | **ns** | **ns** | **ns** | **+** |  | **ns** |  |  | **ns** |  |  | **ns** |  | **ns** |  | **ns** | **ns** | **+** | **ns** |  |  | **+** |  |  | **+** |  |  | **+** | **5** |
| Thrombocytopenia |  | **+** |  | **+** | **+** |  | **ns** |  | **ns** |  |  | **ns** | **ns** |  |  |  |  |  |  |  |  | **+** |  |  | **ns** |  | **+** |  | **5** |
| Abnormal clotting | **ns** | **+** | **ns** |  |  |  |  |  | **+** | **+** | **+** |  |  |  |  |  |  |  |  | **nr** |  |  |  |  |  | **ns** |  |  | **4** |
| Anemia |  |  |  |  |  |  |  |  | **+** |  |  |  |  |  |  |  |  |  |  |  |  |  |  |  | **+** |  | **+** | **+** | **4** |
| Blood pH |  |  | **ns** |  |  |  |  |  |  |  |  | **+** |  |  |  |  |  |  |  |  |  | **+** |  |  |  |  |  |  | **2** |
| Calcium |  |  |  |  |  |  |  |  |  |  |  |  |  |  |  |  |  |  |  |  |  |  |  |  |  |  | **+** | **+** | **2** |
| CRP | **ns** |  |  |  |  |  |  |  |  |  |  |  |  |  |  |  |  |  |  |  |  |  |  |  |  |  | **+** | **+** | **2** |
| Hematocrit |  | **ns** |  |  |  |  |  |  | **ns** |  |  | **+** |  |  |  |  |  |  |  |  |  |  |  |  |  |  |  | **+** | **2** |
| Hypoalbuminemia |  | **ns** |  |  |  |  |  |  |  |  |  |  | **ns** |  |  |  |  |  |  |  |  |  |  |  | **ns** |  | **+** | **+** | **2** |
| Lactate |  | **+** |  |  |  |  |  |  |  |  |  |  |  |  |  |  |  |  |  |  |  |  |  |  |  |  |  | **+** | **2** |
| LDH |  |  |  |  |  |  |  |  |  |  |  |  |  |  |  |  |  |  |  |  |  |  |  |  |  |  | **+** | **+** | **2** |
| Sodium |  |  |  |  |  |  |  |  |  |  |  | **+** |  |  |  |  |  |  |  |  |  |  |  |  |  |  |  | **+** | **2** |
| Bicarbonate |  |  |  |  |  |  |  |  |  |  |  |  |  |  |  |  |  |  |  |  |  |  |  |  |  |  |  | **+** | **1** |
| Pro-calcitonin |  |  |  |  |  |  |  |  |  |  |  |  |  |  |  |  |  |  |  |  |  |  |  |  |  |  |  | **+** | **1** |
| Uric acid |  |  |  |  |  |  |  |  |  |  |  |  |  |  |  |  |  |  |  |  |  |  |  |  |  |  |  | **+** | **1** |

* **+** indicates an increased risk of mortality associated with prognostic factor / - indicated a decreased risk of mortality associated with prognostic factor
* fail – failure / resp – respiratory / LOC level of consciousness
* ns – not significant / nr – level of significance not reported / blank space indicates variable not examined
* ICU – intensive care unit
